# Impact of Trichloroethylene Exposure on the Microbial Diversity and Protein Expression in Anaerobic Granular Biomass at 37°C and 15°C

**DOI:** 10.1155/2012/940159

**Published:** 2012-11-08

**Authors:** Alma Siggins, Anne-Marie Enright, Florence Abram, Catherine Botting, Vincent O'Flaherty

**Affiliations:** ^1^Microbial Ecology Laboratory, Department of Microbiology, School of Natural Sciences, National University of Ireland, Galway, Ireland; ^2^Functional Environmental Microbiology, Department of Microbiology, School of Natural Sciences, National University of Ireland, Galway, Ireland; ^3^BSRC Mass Spectrometry Facility, University of St Andrews, St Andrews KY16 9ST, UK

## Abstract

Granular biomass from a laboratory-scale anaerobic bioreactor trial was analysed to identify changes in microbial community structure and function in response to temperature and trichloroethylene (TCE). Two bioreactors were operated at 37°C, while two were operated at 15°C. At the time of sampling, one of each temperature pair of bioreactors was exposed to process failure-inducing concentrations of TCE (60 mg L^−1^) while the other served as a TCE-free control. Bacterial community structure was investigated using denaturing gradient gel electrophoresis (DGGE) and 16S rRNA gene clone library analysis. Temperature was identified as an important factor for bacterial community composition, while minor differences were associated with trichloroethylene supplementation. Proteobacteria was the dominant phylum in all bioreactors, while clone library analysis revealed a higher proportion of Bacteroidetes-, Chloroflexi-, and Firmicutes-like clones at 15°C than at 37°C. Comparative metaproteomics in the presence and absence of TCE was carried out by two-dimensional gel electrophoresis (2-DGE), and 28 protein spots were identified, with putative functions related to cellular processes, including methanogenesis, glycolysis, the glyoxylate cycle, and the methyl malonyl pathway. A good agreement between metaproteomic species assignment and phylogenetic information was observed, with 10 of the identified proteins associated with members of the phylum Proteobacteria.

## 1. Introduction

Anaerobic digestion (AD) is a sequential and cooperative microbial process, employed in engineered ecosystems for the treatment of wastes and wastewaters and for the production of biogas from biomass and organic residues [1]. Low-temperature operation of laboratory-scale anaerobic digesters has been proven feasible as a cost-effective alternative to traditional mesophilic operating temperatures for a wide range of wastewater types [2, 3]. At all applied temperature ranges, AD relies on the appropriate combination of a variety of microorganisms; complex syntrophic interactions between archaeal and bacterial species are essential for the complete degradation of organic compounds to methane [4]. In the past two decades, the nature of the microbial communities involved in low temperature AD has come under closer scrutiny, with the recognition that greater understanding of the potential and limitations of the microbial consortium could aid in process optimisation. For example, Enright et al. [5] demonstrated that a shift in methanogenic community structure observed by terminal restriction fragment length polymorphism (TRFLP) corresponded to increased hydrogenotrophic activity, while Bialek et al. [6] used statistical analysis (moving window/nonmetric multidimensional scaling) of quantitative polymerase chain reaction (qPCR) data to visualise shifts in the methanogenic communities that could be attributed to bioreactor configuration. Both of these studies, as is traditional for investigation of the microbial communities underpinning the process of anaerobic digestion, focused on the methanogens, a group of anaerobic archaea involved in the conversion of acetate and hydrogen to methane [7]. The low biodiversity associated with methanogens in AD has facilitated the generation of functional and process-relevant information based on methanogenic community structure [8]. 

We have previously documented the response of the methanogenic community within anaerobic granular biomass to the presence of trichloroethylene (TCE; [9]). TCE is a potentially carcinogenic and mutagenic compound [10], which is routinely employed in the cleaning and metal degreasing industries, and can be completely dechlorinated by the process of anaerobic digestion [11]. Our previous study[9] investigated the impact of influent TCE concentrations on the stable operation of expanded granular sludge bed bioreactors at 37°C and 15°C. In order to explore the observed decrease in operational performance noted at both temperatures in response to an influent TCE concentration of 60 mg L^−1^, specific methanogenic activity (SMA) and toxicity batch assays, in addition to qPCR analysis of the methanogenic community, were undertaken [9]. We determined that changes in the methanogenic community in response to TCE were not sufficient to result in the observed process failure, while temporal sampling revealed that changes in temperature resulted in a higher impact on the methanogenic population structure [9]. Specific methanogenic activity and toxicity assays suggested that acetoclastic methanogens were reversibly inhibited by the presence of TCE and/or its degradation derivatives, while competition by dechlorinating organisms may have limited the availability of hydrogen for hydrogenotrophic methanogenesis [9]. Although our study [9] addressed the response of the archaeal community to the presence of TCE, no changes that could definitively account for bioreactor failure at that TCE concentration were identified. Conclusions from that study were based on metabolic groups rather than specific organisms, and disparities in results between molecular and physiological data were observed [9]. Consequently, this study attempts to further investigate the structural response of the bacterial domain, and the functional response of the overall microbial community.

This study investigated the impact of TCE on both the bacterial community structure (using DGGE and 16s rRNA clone library) and the microbial community function (using 2-DGE based metaproteomics) within AD bioreactors operating at 37°C and 15°C.

## 2. Materials and Methods

### 2.1. Source of Biomass

Anaerobic granular sludge originating from four expanded granular sludge bed (EGSB) bioreactors was investigated. All bioreactors (R1–R4) were utilised for the treatment of a volatile fatty acid (VFA)-based wastewater. R1 and R2 were operated at 37°C, while R3 and R4 were operated at 15°C, with R1 and R3 supplemented with increasing concentrations of trichloroethylene (TCE; 10–60 mg L^−1^). The prepared influent was stored in a closed system to prevent volatilisation of TCE; a nitrogen filled gas bag was used to equilibrate the pressure as the influent was pumped from the storage container to the bioreactor. The bioreactor trial and associated performance data are presented in detail in Siggins et al. [9]. Biomass was sampled from the bioreactors on day 235, when influent TCE concentration of R1 and R3 were 60 mg L^−1^. 

### 2.2. Volatile Fatty Acid (VFA) Analysis

Analysis of VFA concentrations of effluent samples collected from R1–R4 throughout the trial were performed by heated (85°C) and agitated headspace, in a Varian Saturn 2000 GC/MS system, with CombiPAL autosampler (Varian Inc., Walnut Creek, CA). Separation was carried out on a Varian Capillary column, CP-WAX 58 (FFAP) CB (25 m length × 0.32 mm internal diameter × 0.2 *μ*m film thickness, Varian). The injector volume was 2 mL and the injector temperature was maintained at 250°C. Helium was employed as the carrier gas, at a flow rate of 1 mL min^−1^. The temperature program was as follows: 50°C (20 s) to 110°C (20 s) at a rate of 2°C min^−1^; from 110°C to 200°C (20 s) at a rate of 20°C min^−1^. The MS-detector was operated in the scan mode in the range of 40–150 m z^−1^ at a temperature of 210°C. Identification of VFAs was achieved by matching chromatographic retention times and spectra of standard compounds (acetic-, butyric- and propionic-acids). Calibration curves of standard VFAs were constructed and used for relative concentration of VFAs in effluent headspace samples, expressed as mg L^−1^.

### 2.3. Specific Methanogenic Activity (SMA) Testing

Biomass samples were screened for metabolic capability using specific methanogenic activity (SMA) tests. These were performed using the pressure transducer technique [12, 13], in which propionate (30 mM), butyrate (15 mM), and ethanol (30 mM) were used as substrates to assay indirect methanogenesis. All assays contained 2–5 g volatile suspended solids (VSS) L^−1^ and were performed in triplicate at the bioreactor operational temperature (R1 and R2 37°C; R3 and R4 15°C). Vials without any substrate were used as controls.

### 2.4. Extraction of Genomic DNA

Total genomic DNA was extracted in duplicate from the four biomass samples using an automated nucleic acid extractor (Magtration 12GC, PSS Co., Chiba, Japan). Granular biomass was finely crushed using a mortar and pestle, and re-suspended in 1x phosphate buffered saline to a ratio of 1 : 4 w/v. A 100 *μ*L aliquot of the biomass suspension was loaded per extraction. Extracted DNA was eluted in 100 *μ*L Tris-HCl buffer (pH 8.0) and stored at −20°C.

### 2.5. Clone Library Analysis of 16S rRNA Genes

Bacterial clone libraries were constructed from the extracted genomic DNA; 16S rRNA genes were PCR-amplified using forward primer 27F (5′-AGA GTT TGA TCC TGG CTC AG-3′; [14]) and reverse primer 1392R (5′ ACG GGC GGT GTG TRC-3′; [15]). Reaction mixtures (50 *μ*L) contained 1.5 mM MgCl_2_, 5 *μ*L 10x NH_4_ buffer (16 mM (NH_4_)_2_SO_4_, 67 mM Tris-HCl (pH 8.8 at 25°C), 0.01% Tween-20), 0.2 mM each dNTP (dATP, dCTP, dGTP, dTTP), 12.5 pmol of each primer, 2 *μ*L template DNA, and 1U *Taq* DNA polymerase. The PCR reactions were carried out using a touchdown PCR under the following conditions: initial denaturation at 95°C for 10 mins, followed by 10 cycles of 95°C for 60 s, annealing at 63°C for 60 s, and extension at 72°C for 120 s, where the annealing temperature was decreased by 1°C per cycle; followed by 20 cycles of denaturation at 95°C for 60 s, annealing at 52°C for 60 s, and extension at 72°C for 120 s, followed by a final 10 min extension at 72°C. Controls containing no DNA were also employed to identify amplification of contaminants, and none was detected. PCR products were ligated into the plasmid vector pCR 2.1-TOPO (Invitrogen) and the hybrid vectors were used to transform* Escherichia coli* TOP 10 competent cells, following the manufacturer's instructions. Transformants were screened using Luria-Bertani (LB) agar plates containing 50 *μ*g mL^−1^ kanamycin. Clone libraries were constructed by growing 96 randomly selected colonies derived from each sample at 37°C overnight in 200 *μ*L LB broth medium containing 50 *μ*g mL^−1^ kanamycin in a 96-well plate.

### 2.6. Amplified rDNA Restriction Analysis (ARDRA)

Ninety-six clones from each library were screened to determine if they contained the appropriately sized insert. Vector-specific M13 forward and reverse primers were used at a concentration of 12.5 pmol, with the other PCR reagents as described previously. PCR conditions were: denaturation at 95°C for 10 min; 30 cycles of: 95°C for 60 s, 55°C for 60 s, 72°C for 60 s; followed by a final extension at 72°C for 10 minutes. Five *μ*L of the resulting PCR products were digested with 0.8 *μ*L of the restriction endonuclease *Hae*III at 37°C for 12–16 hours. The resulting DNA fragments were resolved by electrophoresis on 3.5% (w/v) high resolution agarose and banding patterns were grouped into operational taxonomic units (OTUs).

### 2.7. Partial 16S rDNA Sequencing, Phylogenetic, and Statistical Analysis

Inserts from clones representing the 52 OTU's identified were sequenced on a Licor gel sequencer using vector specific M13 primers (MWG Biotech, Germany). Sequences from this study were aligned with 16S rRNA gene sequences retrieved from BLASTn and the RDP using Clustal X [16], and the phylogenetic inference package Paup* 4.0b8 was used for all phylogenetic analysis [17]. The resulting partial 16S rRNA gene sequences were deposited in the GenBank database under the accession numbers HM749844–HM749879. The Simpson index of diversity (1 − *D*) was calculated using the Primer6 software in order to compare the bacterial diversity of the four biomass samples as revealed by clone library analysis, using the algorithm (*D* = Σ(*n*(*n* − 1)/*N*(*N* − 1)), where *n* is the number of individuals belonging to a species in any given sample and *N* is the total number of individuals present in any given sample [18]. A Simpson's diversity index close to 1 means that the sample is highly diverse [18]. 

### 2.8. Denaturing Gradient Gel Electrophoresis (DGGE)

DGGE analysis of bacterial 16S rRNA genes extracted from the four samples was carried as follows: initial PCR amplification used the primers 341F (5′-CCT ACG GGA GGC AGC AG-3′ [19]) and 517R (5′-ATT ACC GCG GCT GCT GG-3′ [19]), with a 40-base pair GC clamp attached to the 5' terminus of the forward primer.

The touchdown PCR program consisted of an initial denaturation at 94°C for 120 s; followed by 10 cycles of 94°C for 30 s, annealing at 65°C for 30 s, and extension at 72°C for 30 s, where the annealing temperature was decreased by 1°C per cycle; followed by 20 cycles of denaturation at 95°C for 30 s, annealing at 55°C for 30 s and extension at 72°C for 30 s, followed by a final 10 min extension at 72°C. A 40 *μ*L aliquot of GC-clamped PCR product was loaded onto a 10% (w/v) polyacrylamide gel containing a denaturing gradient of 30–70% (where 100% denaturant contained 7 M urea, 40% formamide) and ran at 60°C and 70 V for 16 h in a D-Code system (BioRad, Hercules, CA). The DGGE gels were ethidium bromide stained and photographed under UV trans-illumination. Seventeen bands were selected for further investigation by sequencing and phylogenetic analysis. Ten of these selected bands were present in all samples and were not affected by either temperature or TCE. Six bands were present only at 15°C (R3 and R4), while one band was present only at 37°C (R1 and R2), with both conditions indicating a temperature-dependent response of the microbial community. Selected bands were excised from the gel using a sterile scalpel blade, resuspended in 200 *μ*L of sterile water, and stored at room temperature for three hours to elute DNA from the gel for use as a PCR template. PCR reactions were performed under the conditions described above and the resulting PCR products were cloned using TOPO TA (Invitrogen). Plasmids from five randomly selected clones per reaction were extracted and 2 *μ*L of plasmid DNA was employed as a template for PCR using the same primers and conditions as described previously. For confirmatory purpose, the products of PCR from plasmid DNA were electrophoresed on a DGGE gel in parallel with the corresponding original PCR product. Plasmids that produced bands that underwent denaturation at the same gradient concentration as the original sample, and thereby migrated the same distance through the gel, were selected and sequenced (MWG Biotech, Germany).

Sequences from this study were aligned with 16S rRNA gene sequences retrieved from BLASTn and the RDP using Clustal X [16], and the phylogenetic inference package Paup* 4.0b8 was used for all phylogenetic analysis [17]. The resulting partial 16S rRNA gene sequences were deposited in the GenBank database under the accession numbers HM749788–HM749804.

### 2.9. Statistical Analysis of DGGE Data

DGGE gels were analysed by creating binary matrices, where-by the presence or absence of bands in each sample were denoted with the numeric values “1” or “0”, respectively. These matrices were used to calculated unweighted pair-group methods using arithmetic averages (UPGMA) similarity dendrograms using the PC-ORD 5.0 statistical package [20]. 

### 2.10. Two-Dimensional Gel Electrophoresis (2-DGE)

Proteins were extracted in duplicate from 50 mL of each granular sludge sample by sonication and subsequently separated by 2-DGE [21, 22]. Briefly, the first dimension consisted of isoelectric focusing (IEF) using 7 cm IPG strips with linear pH gradients (pH 4 to 7; Amersham). The second dimension polyacrylamide (12% w/v) gels were run in pairs along with molecular weight markers with a range of 10–225 kDa (Broad Range Protein Molecular Markers, Promega). Gels were stained overnight in GelCode 135 Blue staining reagent (Pierce) and then destained in deionised, distilled water for several hours. Twenty four gels were run corresponding to two duplicate independent extractions and three technical replicates of four samples. Gel images were processed and analysed with PDQuest-Advanced software, version 8.0.1 (BioRad). Spot counts were obtained using the spot detection wizard enabling the Gaussian model option and data normalisation was performed using the Local Regression Model, as recommended by the manufacturer. Ratios of spot intensities were determined in the presence and absence of TCE at both 37°C and 15°C. Protein expression ratios greater than two-fold were considered significant. Proteins deemed of interest were excised from the gels and identified using nanoflow liquid chromatography-electrospray ionization tandem mass spectrometry (nLC-ESI-MS/MS), as previously described [21, 22]. The MS/MS data were analysed using the Mascot 2.2 search engine (Matrix Science, London, UK) against the NCBInr database (04 March 2010, 10,530,540 sequences) with no species restriction. Positive protein identification was based on two criteria: a Mascot Mowse score of >52 (95% confidence level) and a minimum detection of two peptides per protein. 

## 3. Results

### 3.1. Specific Methanogenic Activity

At R1 and R3 influent TCE concentrations of 60 mg L^−1^, the SMA against propionate, butyrate, and ethanol was generally lower for both TCE-supplemented bioreactors (R1 and R3) than their corresponding controls (R2 and R4; Table 1). For each of the three substrates tested, the activity of the TCE-supplemented bioreactors was higher at 37°C (R1) than at 15°C (R3; Table 1), with the same trend observed for biomass originating from the control bioreactors, with activity of R2 (37°C) higher than that of R4 (15°C) for all three substrates utilised in this assay (Table 1).

### 3.2. Clone Library Analysis of 16S rRNA Genes

**Figure 1:**
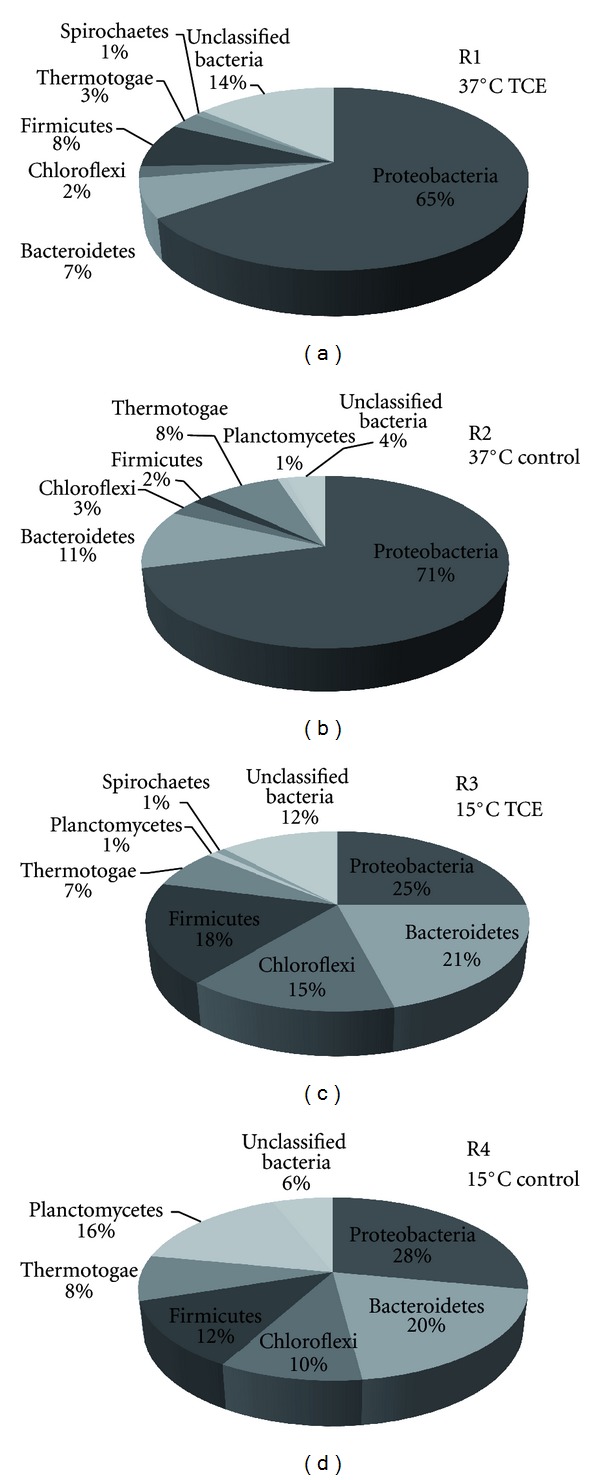
Diversity of bacterial clones obtained from 16S rRNA gene clone libraries. R1: 37°C TCE-supplemented; R2: 37°C control; R3: 15°C TCE-supplemented; R4: 15°C control.

ARDRA was carried out on a total of 354 clones, and several temperature-related, but apparently TCE-independent, changes in the bacterial community structure were observed (Figure 1). For example, although clones related to the phylum Proteobacteria were dominant in all bioreactors, the proportion of Proteobacteria-like clones was higher in both R1 and R2 (37°C, with and without TCE, resp.) than in R3 and R4 (15°C, with and without TCE, resp.), while the opposite was true of Bacteroidetes-, Chloroflexi-, and Firmicutes-like clones (Figure 1). The 15°C biomass samples showed higher species diversity than at 37°C, and Simpson's diversity indices were: R1 (37°C TCE) 0.7032; R2 (37°C control) 0.6384; R3 (15°C TCE) 0.8459; R4 (15°C control) 0.8462.

### 3.3. DGGE

**Figure 2:**
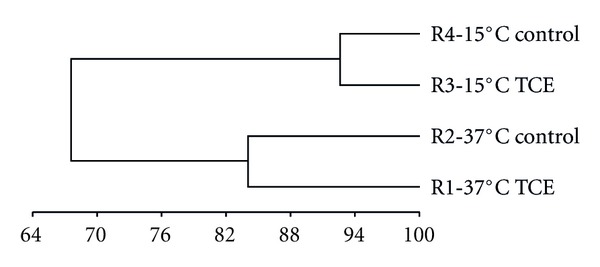
Bacterial UPGMA cluster analysis of 16S rDNA fragments generated from DGGE banding profiles. Percent similarity calculated by (1−Sorensons (Bray-Curtis) distance measurement) ∗ 100. R1: 37°C TCE-supplemented; R2: 37°C control; R3: 15°C TCE-supplemented; R4: 15°C control.

UPGMA analysis of PCR-DGGE data demonstrated that the bacterial community of the biomass statistically clustered by bioreactor operational temperature, with the 37°C bacterial communities (R1 and R2) demonstrating >80% similarity, and the 15°C bioreactors (R3 and R4) demonstrating >90% similarity, irrespective of TCE exposure (Figure 2). Furthermore, the greatest difference in the bacterial community structure of these samples was observed between the 37°C and 15°C bioreactor clusters, which established temperature as a stronger driving force in bacterial community diversity than TCE (Figure 2).

**Figure 3:**
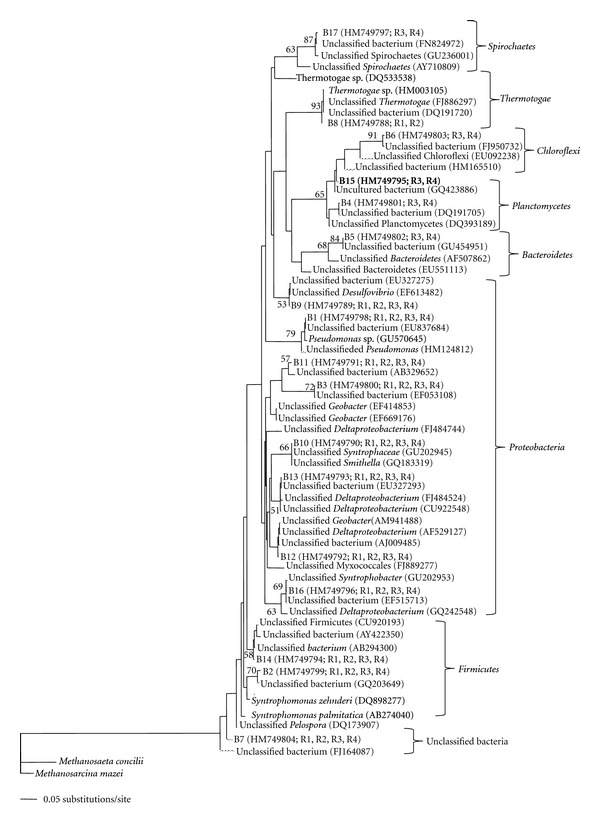
Phylogeny of bacterial sequences obtained by DGGE from R1–R4 biomass, calculated using the Kimura-2 algorithm and the neighbour-joining method [23]. Bootstrap replicates (total 100 replicate samplings) supporting the branching order are shown at relevant nodes. Accession numbers and the bioreactor biomass containing the respective bands are given in parenthesis. R1: 37°C TCE-supplemented; R2: 37°C control; R3: 15°C TCE-supplemented; R4: 15°C control.

Phylogenetic analysis of the seventeen DNA fragments excised from the DGGE gel allowed the identification of organisms within the bacterial community (Figure 3). Of these seventeen bands, ten were present in all samples, of which nine were identified as being closely associated with: *Pseudomonas* (B1), *Syntrophomonas *(B2), *Geobacter *(B3 and B11), *Desulfovibrio *(B9), Syntrophaceae (B10), Myxococcales (B12), Deltaproteobacteria (B13), and Firmicutes (B14), while B7 did not group with any classified bacterial phylum (Figure 3). 

Six bands were only detected at 15°C (R3 and R4) and were related to: Planctomycetes (B4), Bacteroidetes (B5), Chloroflexi (B6 and B15), Deltaproteobacteria (B16), and Spirochaetes (B17); while only B8 was present exclusively at 37°C (R1 and R2) and was identified as a Thermotogae-like species (Figure 3). Overall, six of the seventeen bands analysed were phylogenetically affiliated with Proteobacteria, (B3, B10, B11, B12, B13, B16) establishing it as the most dominant phylum (Figure 3). No bands were observed on DGGE gels that were differentially detected due to the presence or absence of TCE. 

### 3.4. Metaproteomics

Ninety-three distinct spots were excised and sequenced for protein identification based on protein expression ratios. Spots were selected so that proteins that were constitutively expressed, induced or repressed in the presence of TCE were all included for analysis. Of these, 46% were positively identified using nLC-ESI-MS/MS. A number of proteins were found to have migrated as several distinct spots, resulting in the positive identification of 27 unique proteins (Table 2).

Twelve proteins were associated with bacterial species, ten of which were members of the phylum Proteobacteria, with the Actinobacteria and Firmicutes each represented by one protein (Table 2). The functions of proteins originating from bacterial species were highly varied. Proteins associated with the metabolism of components of the influent wastewater, namely acetate (phosphate acetyltransferase) and ethanol (alcohol dehydrogenase), were identified in all samples, and were affiliated with the Proteobacteria (Table 2).

Five proteins were identified that could be involved in the degradation of glyoxylate (Table 2). Glyceraldehyde-3-phosphate dehydrogenase, an enzyme of the glycolytic pathway, was detected in all samples (Table 2), indicating that the glycolytic pathway appears to be active under all of the conditions investigated. In addition, there is evidence of activity of the methyl-malonyl pathway, as methyl malonyl-CoA mutase was detected in all samples, and was upregulated ca. 24-fold in the presence of TCE at 37°C (Table 2). Succinyl-CoA synthetase, associated with the production of succinyl-CoA, which is required for the methyl malonyl pathway, was identified in both 37°C samples, but not at 15°C (Table 2). Malate dehydrogenase, a protein of the glyoxylate cycle that converts malate to oxaloacetate, was also identified in all samples, but was downregulated 3-fold in the presence of TCE at 15°C (Table 2). Oxaloacetate, in turn, can be converted to aspartate, which can lead to the production of L-homocysteine via the formation of O-acetyl-L-homoserine by O-acetylserine sulfhydrylase, an enzyme which was detected in all samples and was found to be upregulated in the presence of TCE at both temperatures (Table 2). O-acetylserine sulfhydrylase was assigned to *Geobacter *sp., known to be involved in TCE dechlorination [24]. 

The remaining nine proteins were assigned to archaeal species belonging to the orders Methanomicrobiales and Methanobacteriales, and to the family Methanosaeta (Table 2). Unsurprisingly, methanogenesis dominated the suggested function of proteins originating from the archaea, with proteins involved in the production of methane from acetate (Acetyl-CoA decarbonylase) and CO_2_ (coenzyme F420 dependent N5, N10 methylenetetrahydromethanopterin reductase) identified in all samples (Table 2). 

In addition, several house-keeping proteins were identified, and included those involved in ATP synthesis and proteolysis, while acetate-CoA ligase was upregulated in the presence of TCE at both temperatures, which would result in increased production of acetyl-CoA (Table 2). Finally, a hypothetical protein of unknown function was detected at 37°C, and affiliated with *Methanospirillum *sp. (Table 2).

## 4. Discussion

**Figure 4:**
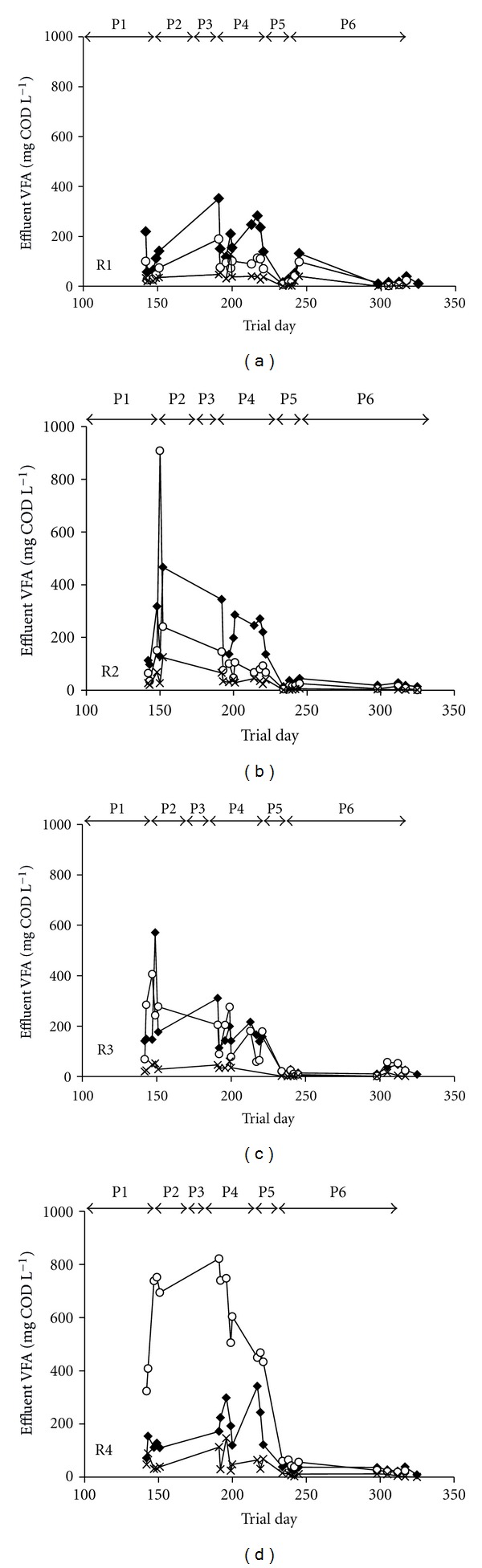
Effluent VFA concentrations of R1–R4: acetic acid (*♦*); propionic acid (○); butyric acid (-x-). R1: 37°C TCE-supplemented; R2: 37°C control; R3: 15°C TCE-supplemented; R4: 15°C control.

Several results indicated a negative response of the bacterial community to the presence of TCE, particularly at the maximum applied concentration of 60 mg L^−1^. For example, the accumulation of measured VFA, particularly propionic acid, was observed during process perturbations following the initial TCE addition and subsequent increases in TCE concentration (Figure 4). Also, SMA assays using indirect methanogenic substrates indicated that on day 235, the activities of propionate, butyrate, and ethanol utilisers were generally lower in the TCE-supplemented bioreactors than in their control counterparts (Table 1). However, analysis of the bacterial community during this study did not reveal any significant changes in the community structure that could be strongly attributed to the process failure observed in both R1 (37°C) and R3 (15°C) at influent TCE concentrations of 60 mg L^−1^ [9]. As was the case of with the archaeal population [9], it would appear that the bacterial community was not structurally affected by the addition of TCE; however the reduced activity of the syntrophic populations may have contributed to process failure.

By employing 16S rRNA gene analysis (DGGE and clone libraries) this study aimed to target the bacterial community structure and identify changes that may have been associated with temperature-and/or TCE-induced process failure. Additionally, metaproteomic analysis of the overall microbial community was carried out in an attempt to identify proteins associated with ongoing functions in the bioreactors, including the reductive dechlorination of TCE.

**Figure 5:**
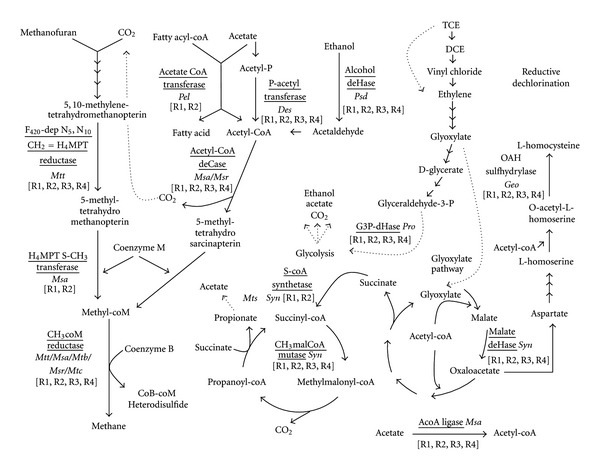
Proposed metabolic pathway for the degradation of VFA and TCE inferred from the metaproteomic data. Enzymes identified in this study are underlined. Species abbreviations are as follows: Mtt Methanothermobacter sp.; Msa Methanosaeta sp.; Mtb Methanobacterium sp.; Msr Methanosarcinales sp.; *Mtc Methanoculleus* sp.; Pel* Pelotomaculum *sp.; Des Desulfuromonas sp.; Psd* Pseudomonas* sp.; Syn* Syntrophobacter *sp.; Pro Propionibacterium sp.; *Geo Geobacter* sp. Enzymes were identified from the bioreactors indicated within square brackets, where R1: 37°C TCE-supplemented; R2: 37°C control; R3: 15°C TCE-supplemented; R4: 15°C control.

The high levels of Proteobacteria*-*like species observed by DGGE and 16S rRNA gene clone libraries in all samples was in keeping with previous studies, which reported that Proteobacteria are commonly amongst the dominant phyla in anaerobic bioreactors [25, 26]. Analysis of the metaproteome confirmed the metabolic activity of Proteobacteria in all samples (Table 2), substantiating the importance of this phylum in the process of anaerobic digestion. Correspondingly, several key Proteobacteria species were identified by one or more of these techniques. For example, propionate-oxidising *Syntrophobacter fumaroxidans* were present in all samples, detected by both bacterial clone library (Figure 1) and DGGE analysis (Figure 3), and were associated with three of the proteins involved in the metabolic pathways outlined in Figure 5. One of these proteins, methyl malonyl CoA mutase, which is involved in the metabolism of propionate (Figure 5), was strongly induced in the presence of TCE at 37°C (Table 2), although SMA assays indicated a lower activity of propionate mediated methanogenesis in the presence of TCE (Table 1). It is possible that production of methyl malonyl CoA mutase increased in response to this metabolic bottleneck, and facilitated the stable production of CH_4_ at 37°C. In addition, as oxidation of intermediate reduced organic compounds such as propionate is energetically unfavourable, *Syntrophobacter* require growth in coculture with methanogens that utilise both hydrogen and formate, such as *Methanospirillum hungatei*, thereby maintaining low concentrations of these oxidation products and allowing energy gain by all organisms involved [27, 28]. qPCR analysis using a primer/probe set specific for the order Methanomicrobiales,to which the *Methanospirillum* belong, quantified 16S rRNA gene numbers of 10^7^–10^9^ copies [gVSS]^−1^ on day 235 [9], while specifically, *Methanospirillum hungatei* was detected by analysis of the metaproteome, and associated with a protein of unknown function (Table 2). 

Clones associated with several bacterial phyla were detected at low levels in various bioreactors, for example: Bacteroidetes*-* and Chloroflexi-like clones were identified in all biomass samples (R1–R4; Figure 1); Spirochaetes-like clones only accounted for 1% of the clones from R1 and R3 biomass and were not detected in either R2 or R4 (Figure 1); and *Planctomycetes*-like clones were detected in R2, R3 and R4 (Figure 1). The phyla *Planctomycetes*, Bacteroidetes,Chloroflexi, and Spirochaeteswere each represented by one DGGE band, which were detected in both 15°C bioreactors, but were not detected at 37°C (B4, B5, B6 and B17, resp., Figure 3). It is possible that PCR amplification for DGGE was biased against the low levels of these phyla in the bioreactors, as the amplification of a more abundant template DNA fragment has been shown to suppress the amplification of a minor template [29]. These phyla have been classically identified within bioreactors, although the exact functions of Bacteroidetes, Chloroflexi, and Spirochaetes are as of yet unknown [30]. As no proteins affiliated with these phyla were identified in any sample (Table 2), the functions of these microbial groups in anaerobic bioreactors could not be determined from this study. The majority of the literature regarding the role of the phylum *Planctomycetes* in granular biomass focuses on the anaerobic ammonium oxidising (ANAMMOX) bacteria [31], which convert nitrate and ammonium directly to dinitrogen gas [32], although none of the known proteins associated with this process were identified in this study (Table 2).

Firmicutes-like species were detected by multiple analytical methods in all of the samples. For example, Peptococcaceae-like clones were detected in each clone library (Figure 1) while metaproteomics detected acetate-CoA transferase in both 37°C bioreactors (Table 2), which was associated with *Pelotomaculum thermopropionicum,* a thermophilic, syntrophic, propionate-oxidising bacterium of the Peptococcaceae family [33]. This species has been shown to grow in coculture with the hydrogenotrophic *Methanothermobacter thermoautotrophicus *[33], which was also found to be active in all biomass samples (Table 2). Furthermore, *Syntrophomonas* species within the Firmicutes phylum were represented by both clone library (Figure 1) and DGGE analysis (Figure 3) in all samples, and have previously been shown to grow syntrophically with *Methanospirillum *species, resulting in the formation of methane [34].

The order Desulfuromonadales, and specifically, *Geobacter* species, were detected in all samples (Figures 1 and 3), and have both been associated with the partial dechlorination of TCE to *cis*-1,2 DCE [24]. A protein associated with *Geobacter* sp., O-acetylserine sulfhydrylase, was upregulated in the presence of TCE, at both 37°C and 15°C (Table 2). O-acetylserine sulfhydrylase is involved in the production of L-homocysteine (Figure 5), which can be further converted to cysteine (involved in protein folding) or methionine (often found to interact with the lipid bilayer of membrane-spanning protein domains; [35]). It is possible then, that an increased production of O-acetylserine sulfhydrylase might lead to an increased level of L-homocysteine as a response to the action of lipophilic TCE, which may associate with the cell membrane, resulting in inhibition of cell membrane ATPases [36]. Interestingly, although *Geobacter* species have been associated with TCE dechlorination [24] and have been detected in this study through both clone library (Figure 1) and DGGE analysis (Figure 3), the use of metaproteomics allowed a suggestion for a specific function for this group to be put forward. 

No proteins specifically associated with the reductive dechlorination of TCE were identified by 2-DGE analysis of the metaproteome (Table 2). One hypothesis is that as the bioreactor influent contained a much higher concentration of VFA's than TCE, it is possible that the TCE concentration induced the production of sufficient enzymes for TCE dechlorination, but was insufficient for detection by 2-DGE. Although the dechlorination of TCE was not monitored during this trial, we have previously reported the successful dechlorination of TCE to DCE (>98%) with a similar experimental design at 37°C and 15°C [37], and at temperatures as low as 7°C [38]. Similar difficulties associated with the detection of specific enzymes involved in bioremediation pathways have been encountered with regards to hydrocarbons [39] and chemical pollutants such as toluene [40]. The absence of a matched metagenomic dataset unquestionably hinders protein identification, for example, previous studies have shown that analysis of the metaproteome of activated sludge by 2-DGE resulted in the identification of 38 proteins [41], while implementation of the 2D-nano-LC method with a metagenomic dataset led to the identification of 5029 proteins [42].

Overall, both clone library and DGGE analysis of the bioreactor bacterial populations identified a divergence from the seed biomass that could be attributed more to temperature than TCE. Specifically, UPGMA analysis of DGGE band diversity revealed that the greatest change in bacterial community development occurred between the bioreactors operating at 37°C and at 15°C (Figure 2), while at both temperatures, TCE resulted in a change of <5% between the TCE-exposed and the control biomass (Figure 2). This supports previous studies, which concluded that while analysis of bacterial population dynamics is important, it is not a reliable indicator of process events, as high levels of dynamism can be observed even during times of functional stability [43, 44]. 

## 5. Conclusions

The following conclusions can now be drawn: (1) changes in the metaproteome could be observed as a function of operational temperature and exposure to TCE; (2) according to DGGE UPGMA data, the major driver for bacterial community structure development in anaerobic bioreactors was temperature, with a limited response to the presence of TCE; (3) the detection of specific function associated proteins (such as TCE reductive dehalogenases) could have been improved by the availability of a metagenomic dataset to assist protein identification.

## Figures and Tables

**Table 1:** Specific Methanogenic Activity (SMA) of biomass sampled from R1 to R4 on day 235 of bioreactor trial, when influent TCE concentrations of R1 and R3 were 60 mg L^−1^ [9] Values shown are expressed as mL CH_4_ gVSS^−1^ day^−1^ and are means of triplicates with std. errors (std. deviation/$\sqrt{n}$, *n* = 3) given in parentheses.

Temperature	Biomass	Propionate	Butyrate	Ethanol
37°C	R1 (TCE)	123 (5)	124 (3)	160 (11)
	R2 (Control)	222 (2)	160 (33)	210 (28)

15°C	R3 (TCE)	82 (5)	14 (1)	51 (5)
	R4 (Control)	164 (1)	23 (2)	48 (11)

**Table 2:** Proteins identified from bioreactor biomass. Ratio = TCE reactor spot intensity: non-TCE control reactor spot intensity, that is, positive values indicate the protein in question was expressed at a higher intensity in the TCE-supplemented reactor, and negative values indicate the protein was expressed at a higher intensity in the control reactor. N.D.: not detected.

Protein	Suggested function	Accession number	Species assignment	Classification	Mascot score	% coverage	37°C ratio	15°C ratio
Acetyl-CoA decarbonylase *β*-subunit	Methanogenesis from acetate	gi 116753609	*Methanosaeta thermophila *	Order Methanosarcinales	253	8	−1.7	−1.2
					171	8	N.D.	1.2
Co-enzyme F420 dependent N5, N10 methylenetetrahydromethanopterin reductase	Methanogenesis from CO_2_	gi 1002717	*Methanothermobacter thermoautrophicus *	Order Methanobacteriales	248	19	−1.4	−1.7
					166	13	−2.6	N.D.
					147	9	1.8	1
Tetrahydromethanopterin-S-methyltransferase H-subunit	Methanogenesis from CO_2_	gi 116754675	*Methanosaeta thermophila *	Order Methanosarcinales	104	2	−3.9	N.D.
Methyl-CoM reductase I *α*-subunit	Methanogenesis	gi 126855	*Methanothermobacter marburgensis *	Order Methanobacteriales	203	8	3.2	−1.6
Methyl-CoM reductase I *α*-subunit	Methanogenesis	gi 284413635	*Methanobacterium sp. *	Order Methanobacteriales	469	25	1.2	−1.9
					396	23	N.D.	−4.6
					237	16	N.D.	−3.7
Methyl-CoM reductase I *α*-subunit	Methanogenesis	gi 47827047	Uncultured Methanosarcinales	Order Methanosarcinales	140	17	2.1	2.8
Methyl-CoM reductase I *β*-subunit	Methanogenesis	gi 126862	*Methanothermobacter marburgensis *	Order Methanobacteriales	393	11	N.D.	−8.3
					223	8	−2.7	N.D.
					148	9	−1.7	−1.5
Methyl-CoM reductase I *β*-subunit	Methanogenesis	gi 126178567	*Methanoculleus marisnigri *	Order Methanomicrobiales	224	5	2.8	1.3
Methyl-CoM reductase I *γ*-subunit	Methanogenesis	gi 116753883	*Methanosaeta thermophila *	Order Methanosarcinales	157	11	−2.7	4.9
Proteasome *α*-subunit	Proteolysis	gi 6093782	*Methanosaeta thermophila *	Order Methanosarcinales	206	16	−5.1	12.5
					151	12	N.D.	1.1
Thermosome	Molecular chaperone	gi 116754081	*Methanosaeta thermophila *	Order Methanosarcinales	272	9	−2.9	N.D. in R4
					273	10	N.D.	1.8
V-type ATP synthase *α*-subunit	ATP synthesis	gi 116754898	*Methanosaeta thermophila *	Order Methanosarcinales	217	10	−1.1	N.D.
					212	10	3.9	N.D.
					199	10	1.9	N.D.
					191	8	−5.6	1.9
Hypothetical protein Mhun-2513	Unknown	gi 88603754	*Methanospirillum hungatei *	Order Methanomicrobiales	1640	46	1	N.D.
Acetate-CoA Ligase	Acetyl-CoA synthesis	gi 116754497	*Methanosaeta thermophila *	Order Methanosarcinales	153	5	3.8	5.9
Iron-containing alcohol dehydrogenase	Ethanol metabolism	gi 77457538	*Pseudomonas fluorescens *	Phylum Proteobacteria	180	3	1.4	N.D.
					159	3	1	−1.7
					122	6	N.D.	1.1
Phosphate acetyltransferase	Acetate metabolism	gi 95930364	*Desulfuromonas acetoxidans *	Phylum Proteobacteria	106	5	−1.4	6.5
Acetate-CoA transferase *β*-subunit	Acetate metabolism	gi 147678377	*Pelotomaculum thermopropionicum *	Phylum Firmicutes	304	18	1.1	N.D.
Glyceraldehyde-3-phosphate dehydrogenase type I	Glycolysis	gi 213959469	*Propionibacterium acnes *	Phylum Actinobacteria	375	17	−2	2
Methyl Malonyl-CoA mutase large subunit	Isomerisation of Succinyl-CoA	gi 116747906	*Syntrophobacter Fumaroxidans *	Phylum Proteobacteria	507	16	23.7	−1.1
Succinyl-CoA synthetase *β*-subunit	Formation of Succinyl-CoA	gi 116749138	*Syntrophobacter Fumaroxidans *	Phylum Proteobacteria	235	15	−1.1	N.D.
Malate dehydrogenase	Glyoxylate pathway	gi 116747908	*Syntrophobacter Fumaroxidans *	Phylum Proteobacteria	383	26	1.8	−3.1
O-acetylserine sulfhydrylase	Homocysteine production	gi 148265277	*Geobacter uraniireducens *	Phylum Proteobacteria	216	10	2.9	3.4
Chaperone protein	Macromolecule assembly	gi 85859704	*Syntrophus aciditrophicus *	Phylum Proteobacteria	555	17	N.D.	2.8
Chaperone protein DnaK	Protein folding	gi 116748478	*Syntrophobacter Fumaroidans *	Phylum Proteobacteria	230	8	−1.5	N.D.
Chaperonin GroEL	Protein folding	gi 148266317	*Geobacter uraniireducens *	Phylum Proteobacteria	620	18	−8.5	3.6
					326	10	3.6	N.D. in R4
Extracellular ligand binding receptor	Receptor activity	gi 116750526	*Syntrophobacter Fumaroidans *	Phylum Proteobacteria	671	28	−1.5	N.D.
					309	17	1.2	−2.9
